# Patient-reported outcome measures in pediatric palliative care—a protocol for a scoping review

**DOI:** 10.1186/s13643-021-01791-6

**Published:** 2021-08-28

**Authors:** Heidi Holmen, Anette Winger, Simen A. Steindal, Charlotte Castor, Lisbeth Gravdal Kvarme, Kirsti Riiser, Kari L. Mariussen, Anja Lee

**Affiliations:** 1 Department of Nursing and Health Promotion, Oslo Metropolitan University, Post Box 4, St. Olavs Place, 0130 Oslo, Norway; 2 Lovisenberg Diaconal University College, Lovisenberggt, 15b, 0456 Oslo, Norway; 3 Faculty of Health Studies, VID Specialized University, Oslo, Norway; 4 Department of Health Sciences, Lund University, Box 157, 221 00 Lund, Sweden; 5 Department of Physiotherapy, Oslo Metropolitan University, Post Box 4, St. Olavs Place, 0130 Oslo, Norway; 6 Division of Paediatric and Adolescent Medicine, Oslo University Hospital HF, Box 4950, Nydalen, 0424 Oslo, Norway

**Keywords:** Pediatric palliative care, Symptom assessment, Patient-reported outcome measures, Health and psychosocial instruments, Scoping review

## Abstract

**Background:**

In pediatric palliative care (PPC), there is a need to involve the child’s voice in situations regarding their symptoms and care needs. Patient-reported outcome measures (PROMs) can be tools to systematically gather data reported from the child or a proxy if the child is not capable to self-report in order to provide the services they need. There has been a rapid development in PROM research the last decade, and there is a need for an overview of current knowledge and experiences in the field. Thus, we aim to explore and summarize what is known from the published research about PROMs in PPC.

**Methods:**

We propose a scoping review following the framework by Arksey and O’Malley and the PRISMA Extension for Scoping Reviews checklist. A systematic search will be performed in the following databases: Medical Literature Analysis and Retrieval System Online (Medline), Excerpta Medica database (EMBASE), Cumulative Index to Nursing and Allied Health Literature (CINAHL), American Psychological Association (APA) PsycInfo, Health and Psychosocial Instruments (HaPI), and Allied and Complementary Medicine Database (AMED). The search will be followed by snowballing to identify key papers and significant researchers for additional citations. Covidence will facilitate the independent review of eligible citations, and data will be extracted and presented descriptively, and thematically analyzed using NVivo.

**Discussion:**

The scoping review suggested in this protocol will identify PROMs which have been proposed in PPC and clarify the experiences with their use. The findings of this review will be relevant for researchers and healthcare personnel caring for children and adolescents in PPC. In addition, by highlighting knowledge gaps about the use of PROMs in PPC, this review will point out future needs within this field of research, which is crucial for improving quality of care in PPC.

**Systematic review registration:**

https://osf.io/yfch2/.

## Background

The field of pediatric palliative care (PPC) includes all children and adolescents living with complex life-threatening or life-shortening conditions. Despite the previous lack of consensus when defining palliative care, and the often interchangeable use of palliative care, hospice care, end of life, and terminal care [1, 2], the definition of PPC seems to be more unified. According to the World Health Organization (WHO), PPC is “the active total care of the child’s body, mind and spirit, and [this] also involves giving support to the family” [3]. Due to life-sustaining technology of modern medicine, especially in high-income countries, an increasing number of children in PPC become young adults [4]. Thus, the patient population is often defined as children, adolescents, and young adults (CAYA; aged 0-25 years) [5]. The vast majority (> 97%) of children in need of PPC live in low- and middle-income countries. Worldwide, children with HIV and congenital malformations represent almost half of the population in need of PPC, followed by children with perinatal conditions, such as extreme prematurity and birth trauma [6]. The latter two also constitute the largest groups in Europe, followed by children with neurologic disease and cancer [7]. Children in PPC suffer a wide range of symptoms during the course of their disease. Although most of them have life-limiting conditions, most of these children become young adults, requiring complex care throughout their childhood and onwards [7].

Although adult palliative care and PPC share the overall aim of quality of life [8], PPC differs from adult palliative care as children move through different development stages while receiving care; they have different communication needs and a stronger dependency on their families [9]. Children often receive treatment that is more aggressive, and they are more frequently hospitalized compared to adults [10, 11]. This is contradictory to the evidence suggesting that homebased PPC is the best way to meet the aim of total care for the child and the family [9, 12–17]. Hospitalization affects the family dynamics because the sick child cannot interact with the rest of the family, or participate in kindergarten, school, or activities as if they were at home [18].

Regardless of where the child receives PPC, the child and family rely on a close and continuous contact with healthcare personnel (HCP) to support their care needs. HCP need specialized knowledge on PPC to meet the complex needs of these children, and ensure quality, continuity, and coordination of care. A specialized palliative care service is associated with increased quality of life for the child [19]. PPC teams need tools for effective communication with each other, as well as with other parts of the healthcare services [20]. At the same time, communication between families and HCP is a core challenge in PPC [21], and systematic data collection (e.g., PROMs) could facilitate this communication and ensure that the relevant needs of the child are in focus.

To enhance quality of PPC and to ensure that the care meets the needs of the child and family, standardized measures for assessing symptoms and care needs can support HCP in their care provision. However, often the care needs are reported on behalf of the child, without directly asking children what they want or desire [22]. The subjective self-report, capturing the patients’ views of their own health, known as “Patient-Reported Outcome” (PRO), is defined as “any report of the patient’s health condition that comes directly from the patient, without interpretation of the patient’s response by a clinician or anyone else” [23]. Patient-reported outcome measures (PROM) is the measure or method to gather these patient reports. In addition, patient-reported experience measures (PREMs) are frequently being applied to capture the patient’s experience or satisfaction when being cared for; however, PRO, PROM, and PREM are often used interchangeably. PROM and PREM are complementary to the objective measures of anthropometrics like blood pressure, height, weight, or various blood analyses. In addition to the self-report of PRO, proxy measures conducted by family members or HCP are particularly relevant for children unable to self-report [24]. One obvious challenge with proxy measures is that PROMs often demand an assessment of subjective experiences, and when these are reported by a proxy, a level of uncertainty is added to the caregiver’s interpretation of the needs of the child [24].

Within PPC, standardized measures to assess the needs of children are not systematically applied [25]. The field with greatest focus has been within quality of life in PPC [26]. However, a systematic review on instruments to measure the impact of interventions on PPC [27] did not find any ideal outcome assessment tool for use in PPC. A systematic review from 2019 addressed the implementation of PRO for children with chronic illness in medical settings and found that in general, implementation of PRO increase attention to psychosocial factors [28]. We have previously assessed experiences with homebased PPC supported by eHealth, and found that systematic assessment of symptoms and needs was regarded important, but underdeveloped [29]. Previous research indicates promising results of implementing PROMs in children with various health challenges, but the relevance of PROMs in PPC has not been systematically assessed.

The main objective for the proposed scoping review is to investigate peer reviewed, published studies on PROMs in PPC to map the nature of current research, to identify and summarize existing knowledge, and to present current evidence gaps [30]. We anticipate that descriptions of PROMs in non-peer reviewed sources would lack a rigorous psychometric development; thus, we will limit our search to peer-reviewed literature.

We anticipate that the results of our scoping review will have implications for clinical practice, future primary research, and future systematic reviews based on the knowledge we identify. However, our findings must be cautiously interpreted, as a scoping review simply aims to map evidence in a field, while a stringent systematic review aims to clearly answer a research question with an appraisal of the risk of bias [30–32].

## Methods

### Design

We will undertake a scoping review, using the framework developed by Arksey and O’Malley [30] following their six steps: (1) Identifying the research question; (2) identifying relevant studies; (3) study selection; (4) charting the data; (5) collating, summarizing, and reporting the results; and (6) consultation exercise. The methodological improvements to this framework as suggested by Levac and colleagues will also guide our study [33]. The Preferred Reporting Items of Systematic Reviews extension for Scoping reviews (PRISMA-ScR) checklist will guide the reporting of our scoping review [34]. A priori protocol was published in the Open Science Framework (OSF) preregistrations (https://osf.io/yfch2/) to enhance replicability and transparency and reduce any publication or reporting bias.

#### Step 1: Identifying the research question

To identify a relevant research question, we engaged in discussions between researchers, clinicians, and user-representatives, all involved in the network “Children in palliative care” (CHIP) initiated and founded at Oslo Metropolitan University, with affiliated national and international members, from a variety of clinical and research institutions, as well as stakeholders and user-representatives. Assessing and managing care needs in PPC was regarded a challenge as identified through clinical and user experiences and previous research within the network [22, 29, 35]. Thus, the recent inclusion of PROMs for children was considered relevant and an area of great importance. Initial scoping searches confirmed the need for investigating the area more closely.

The research question is: What is known from the published, peer reviewed literature about PROMs in PPC?

#### Step 2: Identifying relevant studies

##### Eligibility criteria

Our eligibility criteria will be guided by the population, concept, and context (PCC) tool [36] as described in Table 1. We aim to include scientific and peer reviewed research reporting on the development, use, or evaluation of PROMs in PPC either in research or in the clinical setting. Often, children receiving PPC will not have the ability to report their symptoms and needs, but in order to assess the available tools in a comprehensive matter, we will include all tools and modes of assessments (objective, proxy, and patient reported). The population includes children aged 0-25 years and their caregivers, as well as involved HCP regardless of whether the child receives PPC within healthcare institutions or through homebased services. We will exclude studies reporting solely on measures for adults, but studies reporting on both adults and pediatric populations will be included, and the relevant measures used for the pediatric sample will be extracted. We will perform searches without restriction on publication year in order to broadly map previous research. The searches will be updated prior to publication. We will include papers reporting on primary studies reported in English, German, or a Scandinavian language as we understand these languages and lack funding to translate papers from other than these languages. Review papers will be excluded.

**Table 1:** Population, concept, and context (PCC)

PCC element	Proposed scoping review target
Population	Children, adolescents, and young adults (CAYA) aged 0-25 years in PPC
Concepts	PROMs to assess symptoms, care needs, and/or burden, reported either by the patient, caregivers or HCP (proxy reports)
Context	The child may be cared for at any level of the healthcare services, at home, or included in a research setting.

##### Information sources and search

To conduct a comprehensive and thorough scoping review, we will systematically search in several databases in order to gather eligible studies. As our objective is within the medical field, we will search in Medical Literature Analysis and Retrieval System Online (Medline), Excerpta Medica database (EMBASE), Cumulative Index to Nursing and Allied Health Literature (CINAHL), American Psychological Association (APA) PsycInfo, Health and Psychosocial Instruments (HaPI), and Allied and Complementary Medicine Database (AMED) as these together cover this field nicely. The search will be developed in Medline using text words and subject headings and applied in close collaboration with a librarian with expertise in systematic searches in medical research databases. A second librarian will review our search strategy using the Peer Review of Electronic Search Strategies (PRESS) checklist [37]. An example of the search string developed in Medline is available in Table 2 and will be adopted to the other databases. We will search both reference lists of included papers and investigate work that is citing our included papers to identify other relevant studies through Google Scholar and through snowballing. Identifying significant researchers in the field can provide additional information as well.

**Table 2:** Proposed search terms developed in Medline

Palliative care*	Palliative Care/OR "Hospice and Palliative Care Nursing"/OR exp Terminal Care/OR Palliative Medicine/OR exp Advance Care Planning/Resuscitation Orders/OR "Right to Die"/Terminally ill/OR (palliative or palliate* or palliating).tw,kf. OR (dying or (right adj2 die) or (die adj2 dignity)).tw,kf. OR "supporti* care".tw,kf. OR ((terminal* or "end stage*" or endstage* or "advanced stage*" or "late stage*") adj3 (disease* or ill* or care* or caring or treatment* or period* or nurs* or Patient*)).tw,kf. OR (eol or "end of life").tw,kf. OR (("life limiting" or "life threatening") adj3 (disease* or condition* or illness*)).tw,kf. OR (DNR or DNAR or DNI or ("do not" adj3 (intubat* or resuscitat*))).tw,kf. OR "comfort measure*".tw,kf. OR (advance*1 adj3 (plan*1 or planning or directive*)).tw,kf. OR hospice*.tw,kf.
Child*	exp *Child/or exp *Infant/or *Adolescent/or *Young Adult/OR exp Intensive Care Units, Pediatric/or (PICU* or NICU*).tw,kf. OR exp Pediatrics/or exp Pediatric Nursing/or (pediatric* or paediatric* or peadiatric*).tw,kf. OR (neonatal* or neo-natal* or neonate* or newborn* or new-born* or infant* or baby or babies or toddler* or child* or childhood or kid or kids or girl or girls or boy or boys or minors or underage* or under-age* or teen* or youth* or youngster* or adolescent* or adolescence or preadoles* or pre-adoles* or juvenil* or puber* or pubescen* or pre-puber* or prepuber* or prepubescen* or pre-pubescen* or (young adj2 (adult* or man or men or woman or women or person* or people))).tw,kf.
Patient-reported outcomes*	Patient Reported Outcome Measures/OR exp Patient Outcome Assessment/OR Self Report/OR (PROM or PROMS or PREM or PREMS).tw,kf. OR (selfreport* or patientreport* or ((self or patient* or proxy or proxies or family or families or caregiver* or parent* or mother* or father* or brother* or sister* or sibling*) adj3 report*)).tw,kf. OR ((nurse* or therapist* or physiotherapist* or physician* or pediatrician* or paediatrician* or neonatologist* or cardiologist* or neurologist* or oncologist*) adj3 report*).tw,kf. OR ("patient oriented" adj3 (outcome* or measure* or assessment*)).tw,kf. OR (("patient centered" or "patient centred") adj3 (outcome* or measure* or assessment*)).tw,kf. OR (patient* adj3 "outcome assessment*").tw,kf.

*Results of the three boxes will be combined with “AND”

#### Step 3: Data selection

When the final search string is tailored and applied in all databases, the results will be imported into Covidence [38] for duplicate removal and screening according to the eligibility criteria. Two reviewers (HH and SAS) will assess the same, initial 10% of the citations after duplicates are removed and discuss the relevance of their suggested sources of evidence and the applicability of the inclusion and exclusion criteria. Then, all researchers will partake in the remaining screening until all citations have been assessed by two independent reviewers. Covidence allows a random assignment of citations to assess in order to ensure a dual assessment of all citations by two independent reviewers. Two researchers (HH and AL) will resolve any conflicts following the initial screening through consensus. Citations relevant for full-text screening will be assessed by two independent reviewers, and again two researchers (HH and AL) will resolve any conflicts following full-text reading citations through consensus. The search process, including reasons for exclusion of full-text citations will be presented using the PRISMA flowchart [34].

#### Step 4: Charting the data

##### Data extraction

Agreement upon relevant data to extract will be achieved through review and discussions. Data extraction discussions will be informed by the review process, as well as the research members’ experience within the research and clinical field of PPC. This way, we aim to achieve a consistency in the following data extraction, and transparency in the final scoping review publication. The following data charting process will be based upon these discussions, and the relevant data to extract will be used to develop an extraction chart. Pairs of researchers will independently extract data to this form, and the content will be quality assured by the lead researcher HH. Data items to extract will include, but are not limited to, author, year and country, name and origin of the relevant measure, mode of delivery of the measure (paper or digital), design, psychometric properties, relevant findings, notes on usability or satisfaction, information regarding license or patent owners, and other relevant data suggested through the prior discussions.

#### Step 5: Collating, summarizing, and reporting the results

##### Analysis

Eligible studies will be transferred from Covidence to the qualitative data analysis software NVivo [39]. Any quantitative findings will be transformed to qualitative text [40]. Inspired by thematic synthesis developed by Thomas and Harden [41], we aim to conduct a line-by-line coding and develop descriptive themes. As scoping reviews do not aim to provide a synthesis of findings, we will not generate any analytical themes to synthesize the data. Thus, the scoping review results will be presented descriptively to provide an overview of existing knowledge and gaps in current evidence alongside the descriptive themes from the thematic synthesis based on the PROMs we identify and the related experiences.

##### Critical appraisal of individual sources of evidence

In line with the framework of Arksey and O’Malley [30] and the PRISMA ScR checklist [34], the methodological quality of the included studies will not be appraised, as we hope to include studies with various methodological design with the aim to map the evidence in a field, rather than assessing the effect of an intervention. It will therefore be challenging to quantify a risk of bias using traditional appraisal tools such as the Cochrane risk of bias. Further, based on previous research and our own experiences, we expect studies to inform our review poorly if their methodological quality is low as the richness of data tends to be dependent on the research quality.

#### Step 6: Consultation exercise

In order to make our findings more useful and relevant for clinical practice, we will present our preliminary findings to users and stakeholders in PPC according to the voluntarily “Consultation exercise” in step six of the framework by Arksey and O’Malley [30]. This will primarily include members of the CHIP network, comprising researchers, healthcare personnel, user-representatives, and stakeholders.

## Discussion

The proposed scoping review will identify measures relevant to apply for researchers and HCP caring for a PPC population and will identify knowledge gaps of utmost importance for clinical care and future research to improve quality of PPC.

To the best of our knowledge, this scoping review will be the first to identify specific measures and experiences with these in a pediatric palliative population in order to enhance their care. The population demands various measures, depending on whether the child is able to complete any assessment, or if a proxy assessment by parents or HCP is required. Thus, the results will be extensive, covering aspects of interest for HCP in close contact with these children, their families, and care teams, as well as researchers striving to inform care and policy makers, as well as future research.

### Limitations

As with all research aiming to identify and assess relevant literature, there are limitations to the search and the likelihood of identifying all relevant citations. Language restrictions might limit the findings, and likewise the lack of standardized terminology within the field of PPC and PROMs literature. We will strive to ensure a stringent, replicable, and exhaustive search and the addition of searches in reference lists and among significant researchers in the field will reduce this vulnerability. The rapid evolvement of PROMs in PPC might also limit our findings, as we have limited possibilities to detect unpublished literature besides what is already identified throughout network and significant researchers.

## Data Availability

The datasets generated in the scoping searches will be made available upon reasonable request.

## Abbreviations

AMED: Allied and Complementary Medicine Database
CHIP: Children in Palliative Care
CINAHL: Cumulative Index to Nursing and Allied Health Literature
EMBASE: Excerpta Medica database
HaPI: Health and Psychosocial Instruments
HCP: Healthcare personnel
MEDLINE: Medical Literature Analysis and Retrieval System Online
PCC: Population, concept, and context
PREMs: Patient-reported experience measures
PRO: Patient-reported outcome
PROMs: Patient-reported outcome measures
PPC: Pediatric palliative care
PRESS: Peer Review of Electronic Search Strategies
PRISMA-ScR: Preferred Reporting Items of Systematic Reviews extension for Scoping reviews
psycINFO: American Psychological Association (APA) PsycInfo
WHO: World Health Organization
